# Decline in Constitutive Proliferative Activity in the Zebrafish Retina with Ageing

**DOI:** 10.3390/ijms222111715

**Published:** 2021-10-28

**Authors:** Ismael Hernández-Núñez, Ana Quelle-Regaldie, Laura Sánchez, Fátima Adrio, Eva Candal, Antón Barreiro-Iglesias

**Affiliations:** 1 Departamento de Bioloxía Funcional, CIBUS, Facultade de Bioloxía, Universidade de Santiago de Compostela, 15782 Santiago de Compostela, Spain; ismael.hernandez@rai.usc.es (I.H.-N.); fatima.adrio.fondevila@usc.es (F.A.); eva.candal@usc.es (E.C.); 2 Departamento de Zooloxía, Xenética y Antropoloxía Física, Facultade de Veterinaria, Universidade de Santiago de Compostela, 27002 Lugo, Spain; ana.quelle@usc.es (A.Q.-R.); lauraelena.sanchez@usc.es (L.S.); 3 Preclinical Animal Models Group, Health Research Institute of Santiago de Compostela (IDIS), 15706 Santiago de Compostela, Spain

**Keywords:** retina, zebrafish, proliferation, secondary neurogenesis, PCNA, pH3, ageing

## Abstract

It is largely assumed that the teleost retina shows continuous and active proliferative and neurogenic activity throughout life. However, when delving into the teleost literature, one finds that assumptions about a highly active and continuous proliferation in the adult retina are based on studies in which proliferation was not quantified in a comparative way at the different life stages or was mainly studied in juveniles/young adults. Here, we performed a systematic and comparative study of the constitutive proliferative activity of the retina from early developing (2 days post-fertilisation) to aged (up to 3–4 years post-fertilisation) zebrafish. The mitotic activity and cell cycle progression were analysed by using immunofluorescence against pH3 and PCNA, respectively. We observed a decline in the cell proliferation in the retina with ageing despite the occurrence of a wave of secondary proliferation during sexual maturation. During this wave of secondary proliferation, the distribution of proliferating and mitotic cells changes from the inner to the outer nuclear layer in the central retina. Importantly, in aged zebrafish, there is a virtual disappearance of mitotic activity. Our results showing a decline in the proliferative activity of the zebrafish retina with ageing are of crucial importance since it is generally assumed that the fish retina has continuous proliferative activity throughout life.

## 1. Introduction

Neurogenesis is the process by which neural progenitor cells give rise to mature neurons and glial cells. Early in development, the central nervous system (CNS) is formed from a highly active neurogenic neuroepithelium. As development progresses, proliferative and neurogenic activities are gradually lost in most CNS regions, and, in postnatal life, neurogenic activity is restricted to specific regions called neurogenic niches [1,2]. Moreover, the presence of postnatal neurogenic activity in the CNS was also progressively lost during vertebrate evolution (reviewed in [3,4,5,6,7,8]). Accordingly, different vertebrate species show different postnatal/adult proliferative and neurogenic rates and different numbers of neurogenic niches in the CNS, which are more abundant in teleost fishes (reviewed in [3,4,5,6,7,8,9]). Some postnatal constitutive and/or inducible (e.g., during regeneration) neurogenic niches are found in the retina of vertebrates. These include the ciliary marginal zone (CMZ), which is a circumferential ring of cells located in the peripheral retina [10,11,12,13,14,15]; the Müller glial cells of the inner nuclear layer (INL) of the central retina [12,16,17,18,19]; the retinal pigment epithelium (RPE; [20,21,22]), a pseudostratified region at the junction between the retina and the ciliary body [23]; and the pigmented and non-pigmented epithelium of the ciliary body [24,25,26,27,28]. The proliferative and neurogenic capacities of each of these retinal neurogenic niches varies in different vertebrate species (reviewed in [19,29,30,31]). In fishes, all retinal cell types, except rod photoreceptors, are generated within the CMZ and incorporated to the most peripheral region of the central retina (so that older cells remain in the central retina and new cells become located successively in more peripheral positions). Instead, rod photoreceptors are continuously generated from Müller glia in the central retina.

Based on studies in teleost species (see Table S1), it is largely assumed that the retina of fishes, in contrast to mammals, has continuous proliferative activity throughout life and that this (together with tissue stretching) is partially responsible for continuous eye growth, even during adulthood. This idea emerges in relevant articles on this topic during the last decades: “Fish retinas differ fundamentally from those of other vertebrates because they continue to grow throughout the life of the animal, both by adding new neurons and by stretching existing retinal tissue” [32]; “In fish and amphibia, retinal stem cells located in the periphery of the retina, the ciliary marginal zone (CMZ), produce new neurons in the retina throughout life” [33]; “The retina of many fish and amphibians grows throughout life, roughly matching the overall growth of the animal. The new retinal cells are continually added at the anterior margin of the retina, in a circumferential zone of cells […]” [34]; “The retinas of lower vertebrates grow throughout life from retinal stem cells (RSCs) and retinal progenitor cells (RPCs) at the rim of the retina” [35]; “In the retina of teleost fish, cell addition continues throughout life involving proliferation and axonal growth” [36], to name a few. However, studies from our group in the sea lamprey, *Petromyzon marinus*, and the catshark, *Scyliorhinus canicula*, revealed the loss of proliferative activity in the retina of adult individuals of these ancient vertebrate groups [37,38]. This raised the possibility that continuous proliferative activity throughout life in the retina was a derived characteristic of modern teleost fishes and not the ancestral character common to all fish groups [38].

Based on our recent work in sharks [38], we decided to revisit the teleost literature on this topic (see Table S1). We observed that assumptions about continuous proliferative and neurogenic activity in the retina of teleost fishes are mainly supported by work on juveniles and young adults, using animals in which the precise age is not indicated or by studies that did not systematically quantify the proliferative activity at different developmental and postnatal stages (see Table S1). In the zebrafish, *Danio rerio*, qualitative assessments of proliferating cells labelled with bromo-deoxyuridine (BrdU) in the CMZ and central retina from embryonic (24 hours post-fertilisation [hpf]) to young adult (6–8 months post-fertilisation [mpf]) stages revealed a sharp decline in the rate of retinal growth between 3 and 4 days post-fertilisation (dpf) and a decline in the rate of cell addition between embryos and young adults [39]. More recently, a thorough study by Van Houcke et al. ([40]; see Table S1) evaluated the relative contribution of cellular addition and tissue stretching to retinal growth in the adult zebrafish from 6 to 48 mpf. By using immunohistochemical staining for proliferating cell nuclear antigen (PCNA), they quantified progenitor cell proliferation in the adult CMZ and found that the neurogenic capacity of the CMZ strongly declines between 6 and 12 mpf and continues at very low rates up to 48 mpf, though new cells continue to be added to the retina throughout life. However, while proliferating cells were also detected in the central retina, cell proliferation was not investigated over time in this area. On the other hand, since PCNA expression can also be detected long after the cell cycle exit and can also indicate DNA repair or cell death (see [41] and references therein), using PCNA alone as a proliferation marker may overestimate the number of cells progressing through the cell cycle, especially at the oldest stages.

To address these issues, we systematically quantified both the number of cells progressing through the cell cycle (immunoreactive to PCNA) and the number of cells undergoing mitosis (immunoreactive to the mitosis marker phosphohistone H3; pH3) in the CMZ and central retina of zebrafish, covering all the major life stages from early developing (2 dpf) to sexual maturation (1.5 to 3 mpf) and ageing (up to 3–4 years post-fertilisation [ypf]). Our results show that there is a progressive loss of proliferative activity in the retina throughout life in zebrafish despite the occurrence of a wave of secondary proliferation during sexual maturation. Importantly, mitotic activity is virtually absent in the retina of old animals.

## 2. Results

**Figure 1:**
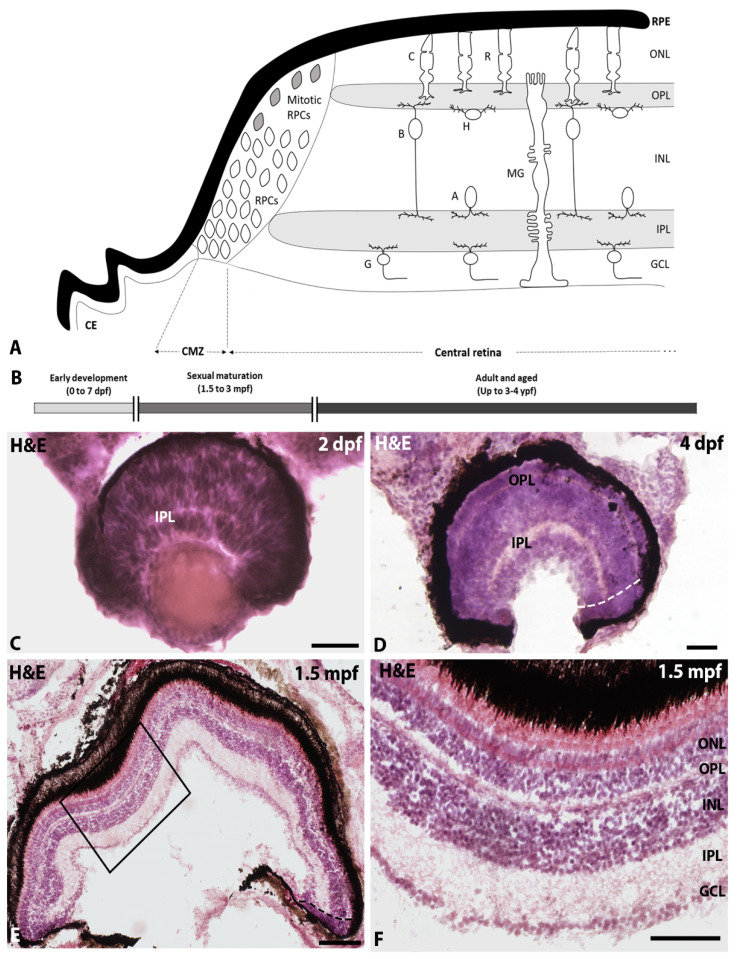
(**A**) Schematic drawing of the mature zebrafish retina showing the ciliary epithelium of the ciliary body (CE), the retinal pigment epithelium (RPE) and the neural retina with two differentiated regions: the CMZ, which contains different types of retinal progenitor cells (RPCs), and the central retina with a layered structure, which contains the outer (OPL) and inner (IPL) plexiform layers and three cell layers [ONL with the nuclei of cone (C) and rod (R) photoreceptors; inner nuclear layer (INL) with the nuclei of bipolar (B), amacrine (A), horizontal (H) and Müller glia (MG) cells; and a GCL with the nuclei of ganglion cells (G)]. (**B**). Timeline of the zebrafish life stages and ages analysed in this study. (**C**–**F**). Hemaetoxylin-eosin-stained transverse sections of the retina of 2 dpf (**C**), 4 dpf (**D**) and 1.5 mpf (**E**,**F**) zebrafish showing the maturation of retinal organization. Dashed lines in (**D**) and (**E**) indicate the limit between the CMZ and the central retina. (**F**) Detail of the central retina squared in (**E**). Scale bars: (**C**,D): 50 µm; (**E**): 200 µm; (**F**): 100 µm.

The zebrafish retina exhibits the typical morphology and structure of the vertebrate retina, with a CMZ located at the retinal margin containing different types of progenitor cells (Figure 1A) and a highly organised central retina (Figure 1A), which can be observed from 2.5 dpf [42], formed by three nuclear layers: the outer nuclear layer (ONL), where the nuclei of photoreceptors are located; the INL, where the nuclei of horizontal, bipolar, amacrine, and Müller glia cells are located; and the ganglion cell layer (GCL), which contains the nuclei of ganglion cells. These cells become connected within two plexiform layers: the outer plexiform layer (OPL) and the inner plexiform layer (IPL).

In this study, we analysed zebrafish of different developmental/life stages: 2, 4, and 7 dpf, 1.5, 2.5, 3, 8.5, and 18–20 mpf and 3–4 ypf. The 2 to 7 dpf period coincides with early zebrafish development (Figure 1B; the zebrafish has a functional retina at about 3 dpf [43]). From 1.5 to 3 mpf, zebrafish are in the process of sexual maturation, and, at 8.5 mpf, they are sexually mature and in peak fertility (Figure 1B). From 18 mpf, the synaptic integrity begins to decline and a gradual increase in the senescence-associated β-galactosidase is observed in the RPE. This senescence marker is detected in the neural retina at 48 mpf [40].

In the 2 dpf animals, the retina did not show the typical layered organisation of a mature vertebrate retina, and it is mainly formed by neuroepithelial cells (Figure 1C). As can be observed in the haematoxylin–eosin-stained sections, the cell nucleus occupies almost the entire cell body in most retinal cells, which is characteristic of proliferating tissues (Figure 1C). From 4 dpf, we observed the typical layered organisation of the central retinal and that the CMZ was progressively reduced and restricted to the most marginal region with ageing (Figure 1D–F).

### 2.1. Changes in Proliferative Activity with Ageing

**Figure 2:**
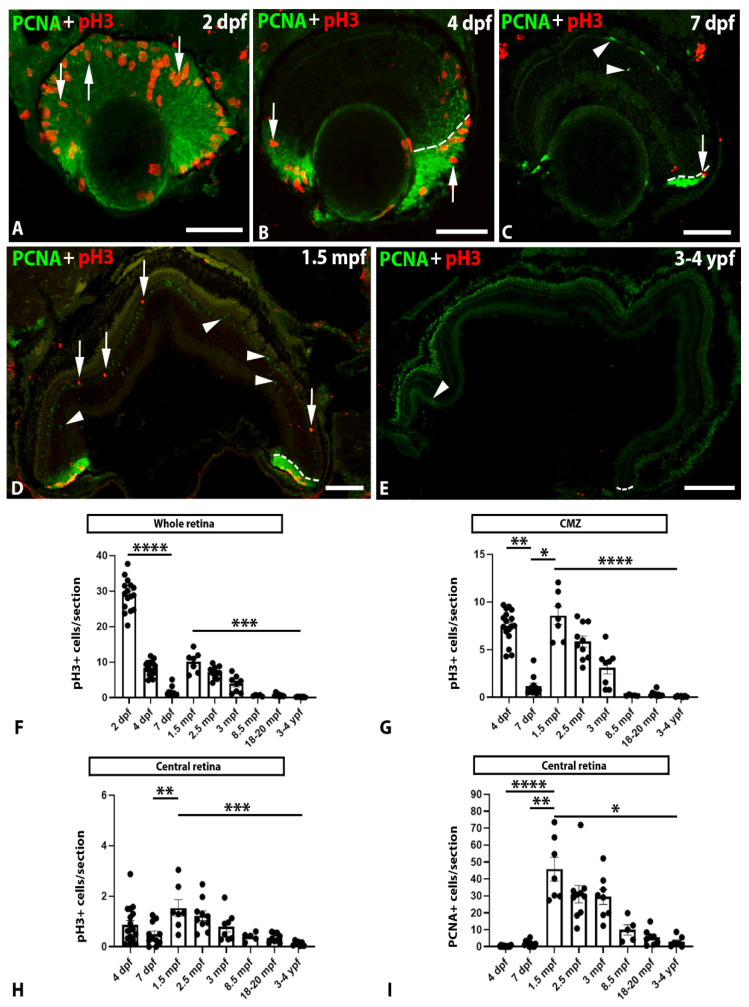
(**A**–**E**). Transverse sections of the retina of 2 dpf (**A**), 4 dpf (**B**), 7 dpf (**C**), 1.5 mpf (**D**), and 3–4 ypf (**E**) zebrafish specimens showing the presence of PCNA (arrowheads) or pH3 (arrows) positive cells. In 2 dpf to 1.5 mpf zebrafish (**A**–**D**), PCNA or pH3 expressing cells were mainly located in the CMZ but also in the central retina, while, in 3–4 ypf, specimens (**E**) pH3+ and PCNA+ cells almost disappeared. Dashed lines in (**B**–**E)** indicate the limit between the CMZ and the central retina. Scale bars: (**A**–**C**): 50 µm; (**D**): 100 µm; (**E**): 200 µm. (**F**). Graph showing significant changes in the number of pH3+ cells/section in the whole retina at different ages (Kruskal-Wallis test, *p* < 0.0001). (**G**). Graph showing significant changes in the number of pH3+ cells/section in the CMZ at different ages (Kruskal-Wallis test, *p* < 0.0001). (**H**). Graph showing significant changes in the number of pH3+ cells/section in the central retina at different ages (one-way ANOVA, *p* < 0.0001). (**I**). Graph showing significant changes in the number of PCNA+ cells/section in the central retina at different ages (Kruskal-Wallis test, *p* < 0.0001). Mean ± S.E.M. data and data on statistical multiple comparisons related to these graphs can be found on File S1. Asterisks indicate different levels of statistical significance: *, *p* < 0.05; **, *p* < 0.01; ***, *p* < 0.001; ****, *p* < 0.0001.

To compare the number of cells progressing through the cell cycle and cells undergoing mitosis in the zebrafish retina at different developmental/life stages, we used double immunofluorescence to detect the expression of PCNA, which is present in proliferating cells during every phase of the cell cycle, peaking from G1 to S and decreasing at G2/M, and pH3, a marker of M-phase cells [44]. Our combinatorial analysis by studying both PCNA and pH3 expression helps to overcome the problem of using PCNA alone as a proliferation marker since the latter can also be indicative of DNA repair or cell death (see Introduction), which can lead to an overestimation of the number of proliferating cells. The number of PCNA+ or pH3+ cells is given as the mean number of cells per retinal section for each animal to allow for a comparison between specimens of different age and size. As expected, no colocalisation of PCNA and pH3 was observed in any of the samples (Figure 2). The number of PCNA+ cells was not quantified in the CMZ because, at some stages (mainly 2 dpf), the high number of positive cells impeded clearly differentiating between the cells individually.

Mitotic (pH3+) cells were mainly observed in the CMZ, and, in this region, most of them were observed in the apical surface, i.e., near the ventricle (Figure 2A–D). However, some ectopic mitoses were also observed in the different layers of the central retina at the different developmental/life stages (Figure 2A,B,D). The pH3+ cells were almost absent in the whole retina of aged specimens (Figure 2E). Most of the cells progressing through the cell cycle (PCNA+) were also located in the CMZ (Figure 2A–E). However, as for pH3+ cells, PCNA+ cells were also present in the different cell layers of the central retina (Figure 2D). The number of PCNA+ cells was highly reduced in the whole retina of aged specimens (Figure 2E).

In the whole retina, the number of pH3+ cells significantly and progressively decreased during early development from 2 to 7 dpf (Figure 2F; File S1). Interestingly, we observed an increase in the mitotic activity of the whole retina at 1.5 mpf (which did not reach the levels of the 2 dpf animals; Figure 2F; File S1). From 1.5 mpf onwards, we observed a significant and progressive decline in the number of mitotic cells with ageing (Figure 2F; File S1). Importantly, mitotic cells were almost absent in the retina of aged specimens (from 8.5 mpf to 3–4 ypf, Figure 2F; File S1).

Very similar trends in temporal expression patterns were observed when looking separately at the number of pH3+ cells per section in the CMZ (Figure 2G; File S1) and central (Figure 2H; File S1) retina or at the number of PCNA+ cells per section in the central retina (Figure 2I; File S1).

Our results indicate that proliferative activity decreases with age in both the CMZ and central region of the zebrafish retina despite the occurrence of a secondary wave of proliferation during sexual maturation (i.e., 1.5 to 3 mpf). Importantly, mitotic activity is virtually absent in aged specimens.

### 2.2. Changes in the Location of Proliferating Cells of the Central Retina with Age

**Figure 3:**
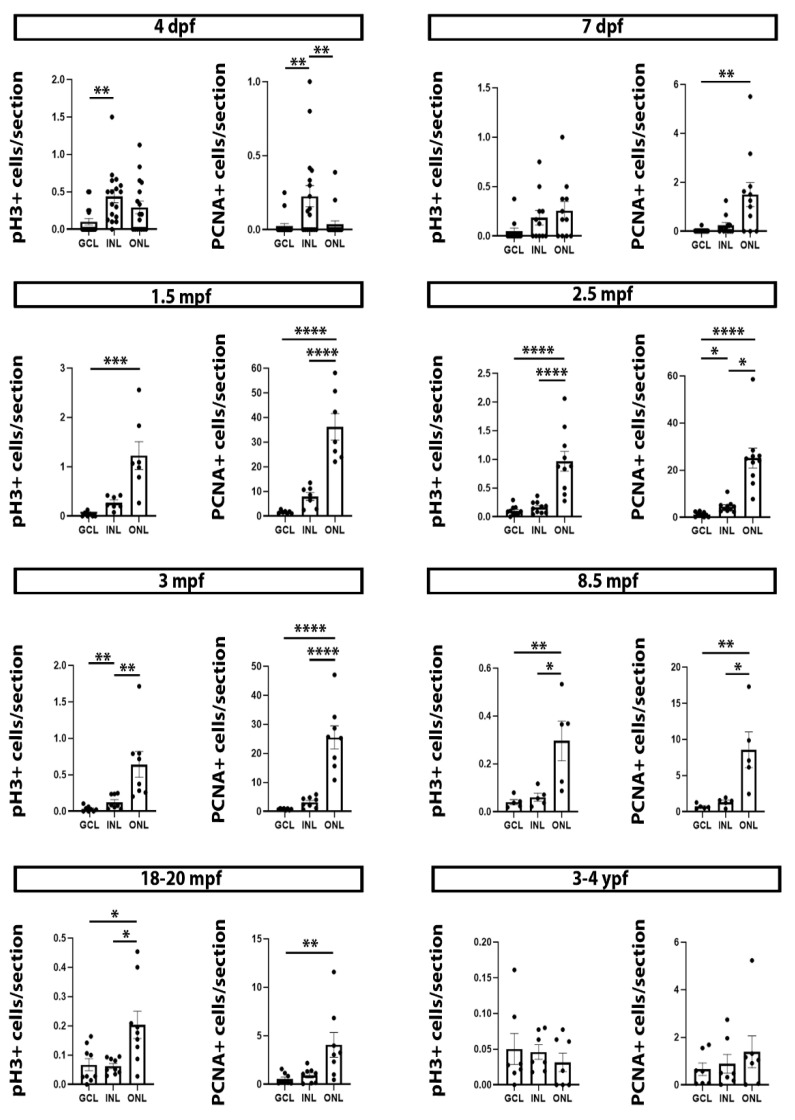
Graphs showing the differential distribution of pH3+ and PCNA+ cells in cell layers of the central retina at different developmental and life stages. 4 dpf specimens: pH3 (Kruskal-Wallis test, *p* = 0.0022), PCNA (Kruskal-Wallis test, *p* = 0.0018). 7 dpf specimens: pH3 (Kruskal-Wallis test, *p* = 0.0655), PCNA (Kruskal-Wallis test, *p* = 0.0024). 1.5 mpf specimens: pH3 (Kruskal-Wallis test, *p* < 0.0001), PCNA (one-way ANOVA, *p* < 0.0001). 2.5 mpf specimens: pH3 (one-way ANOVA, *p* < 0.0001), PCNA (Kruskal-Wallis test, *p* < 0.0001). 3 mpf specimens: pH3 (one-way ANOVA, *p* = 0.0009), PCNA (one-way ANOVA, *p* < 0.0001). 8.5 mpf specimens: pH3 (one-way ANOVA, *p* = 0.0055), PCNA (one-way ANOVA, *p* = 0.0038). 18–20 mpf specimens: pH3 (Kruskal-Wallis test, *p* = 0.0121), PCNA (Kruskal-Wallis test, *p* = 0.0084). 3–4 ypf specimens: pH3 (Kruskal-Wallis test, *p* = 0.4814), PCNA (Kruskal-Wallis test, *p* = 0.8584). Mean ± S.E.M. data and data on statistical multiple comparisons related to these graphs can be found on File S2. Asterisks indicate different levels of statistical significance: *, *p* < 0.05; **, *p* < 0.01; ***, *p* < 0.001; ****, *p* < 0.0001.

We separately quantified the numbers of pH3+ and PCNA+ cells in the cell layers of the central retina (GCL, INL, and ONL) at the different developmental and life stages (Figure 3; File S2). Interestingly, in early developing 4 dpf specimens, the numbers of pH3+ and PCNA+ cells were significantly higher in the INL than in the GCL or ONL (Figure 3; File S2), whereas, from 7 dpf to 18–20 mpf (both included), the numbers of pH3+ and PCNA+ cells were significantly higher in the ONL than in the GCL or INL (Figure 3; File S2). As can be observed in Figure 3, this difference is highly significant during sexual maturation (1.5 to 3 mpf). In the oldest animals (3 to 4 ypf) the very few pH3+ or PCNA+ cells located in the central retina did not show differential distribution in the cell layers (Figure 3; File S2). These results suggest that the progenitor cells that remain in the mature central retina after early development could mainly contribute to the production of the mature cell types of the ONL.

## 3. Discussion

As indicated in the Introduction, it is largely assumed that the retina of fishes shows continuous and active proliferation and neurogenesis throughout life. This assumption is based on previous work in teleost models in which the presence of proliferating cells was only studied in juveniles or young adults, in animals in which the precise age was not defined or known by the authors of the study, or without performing quantitative comparisons between all life stages or ages (see Table S1). This feature of throughout-life neurogenesis does not apply to lampreys or cartilaginous fishes, in which proliferative activity is virtually absent in adult animals [37,38]. Moreover, some of the previous studies on teleost fishes provided qualitative descriptions that also suggested a loss of proliferating cells with age (see Table S1). For example, Johns and Fernald [45] reported that, when studying African cichlid and goldfish juveniles and adults, the dividing cells in the ONL were easier to demonstrate in younger fish. In zebrafish, Marcus et al. [39] also indicated that the number of BrdU labelled cells was greater in the CMZ and central retina of embryos than in young adults (6–8 mpf). A recent study by Van Houcke et al. [40] showed a decline in the cell proliferation (PCNA+ cells) in the zebrafish CMZ from 6 to 48 mpf. However, the proliferation within the central retina of zebrafish was not quantified over time. Besides, the assessment of progenitor cell proliferation relied only on PCNA expression, which, despite its use, can lead to the overestimation of proliferation in aged animals (see the Introduction).

Here, we obtained quantitative data comparing the cell cycle progression (PCNA+ cells/section) and mitotic activity (pH3+ cells/section) in both the CMZ and the central zebrafish retina at different ages and covering all major life stages. Our results show that there is a drastic decline in proliferative activity from 2 to 7 dpf, a continuous reduction in the number of proliferating cells in sexually maturing and old animals, and that cells undergoing mitosis are virtually absent in old animals. This is in good agreement with previous reports of a drastic decrease in cell proliferation in early larvae (between the 3 dpf and 4 dpf; [39]) and with reports of a significant proliferation decrease in early adulthood (6–12 mpf), with very reduced proliferation rates at the mid (18–24 mpf) and late (36–38 mpf) adult stages [40]. As expected, the number of PCNA+ cells reported in the CMZ by Van Houcke et al. [40] was higher than that of pH3+ cells in this region at similar life stages (present results) since the latter are only a fraction of the number of cells progressing through the cell cycle. Our quantitative results in zebrafish reveal a similar pattern to that reported in our work in lampreys and sharks showing a loss of the proliferating and mitotic cells in the adult retina [37,38]. However, it seems that retinal proliferative activity is maintained at a higher rate in adult zebrafish than in adult lampreys (no PCNA+ cells; [37]) or sharks (very few PCNA+ cells and almost no pH3+ cells; [38]).

Unlike previous reports on the proliferation in the zebrafish retina, our systematic analysis allowed unveiling the occurrence of a secondary wave of proliferation during sexual maturation (i.e., from 1.5 to 3 mpf) affecting both the CMZ and the central retina. Only a study by Bernardos et al. [17] provided a qualitative description indicating that BrdU+ cells in the ONL were observed in higher numbers in 1 to 2 mpf animals than in 7 dpf animals. This increase in the cell proliferation at 1.5 mpf (present results) did not reach the levels of the early developing (2 dpf) period, but it was significantly higher than in the 7 dpf specimens. This secondary wave of proliferation could be related to an earlier peak of cell death that occurs in the retina (especially in the ONL) of 7 dpf zebrafish [46]. This increase in cell proliferation could allow for the replacement of the cells lost during this critical period in which fish transition from acquiring nutrition from their yolk to active feeding. This secondary wave of proliferation could also be related to retinal adaptations that might be needed for sexual behaviours, especially since the integration of multi-sensory information between olfaction and vision has been implicated in mating-like behaviours in zebrafish [47]. However, current data have only implicated dopaminergic interplexiform and retinal ganglion cells in this olfacto–visual centrifugal pathway [47], which would not explain why proliferation and neurogenesis are needed in the ONL (see below). Future studies should decipher whether this secondary wave of proliferation is only needed to replace lost retinal cells or if it is related to retinal adaptations needed for sexual (or adult) behaviours in zebrafish.

By looking at the distribution of the proliferating/mitotic cells in the cell layers of the central retina at different ages, we observed that, in early developing 4 dpf specimens, the numbers of pH3+ and PCNA+ cells were higher in the INL, whereas, in older animals, they were more abundant in the ONL. Previous studies have shown that the progenitor cells of the central retina (Müller glia) in juvenile/adult goldfish [45,48,49] and in juvenile zebrafish [17,50,51,52] generate new rods, which indicates that the higher cell proliferation and mitotic activity we observed in the ONL of juvenile and adult zebrafish is related to rod generation. As far as we are aware, the generation of other retinal cell types from the INL and ONL progenitors of the un-injured juvenile/adult teleost retina has not been reported, although injury-induced proliferating Müller glial cells can regenerate all the retinal cell types, including cones [17,50,51,52], which has led to the suggestion that the neuronal progenitors produced by Müller glia are multipotent and can revert to an earlier lineage under the influence of certain microenvironmental signals [17,50,51,53]. The zebrafish retina presents five main types of photoreceptors (four cones and one rod), and the five types of photoreceptors are generated during early development [54,55]. Perhaps one or more of these photoreceptor types are specifically needed for mating/courtship/adult behaviours and could be generated in extra numbers during sexual maturation, which could explain the secondary wave of cell proliferation we detected in zebrafish juveniles. Since, during courtship and spawning, female zebrafish discriminate between the sexes using visual cues in which the male yellow colouration is critical [55], it is tempting to hypothesise that specific cones might be needed at this life stage. However, to our knowledge, microenviromental signals other than retinal injury driving cone generation from progenitors in the central retina have not been experimentally assessed. Future work should attempt to study whether cones could also be generated from these dividing progenitor cells of the central retina, especially during the previously undetected secondary wave of proliferation at the time of sexual maturation.

## 4. Materials and Methods

### 4.1. Animals

2 dpf (*n* = 16), 4 dpf (*n* = 17), 7 dpf (*n* = 11), 1.5 mpf (*n* = 7), 2.5 mpf (*n* = 10), 3 mpf (*n* = 8), 8.5 mpf (*n* = 5), 18–20 mpf (*n* = 9), and 3–4 ypf (*n* = 7) zebrafish (*Danio rerio*) specimens were used in this study. Zebrafish were kept in aquaria under standard conditions of temperature (28 °C), light cycle (14 h of light and 10 h of darkness), and pH (7.0) until use for experimental procedures. All juvenile/adult fish were kept at a density of 3 fish/litre, and food supply was under strict standardised control to avoid differences in animal size in each of the experimental groups [40]. Juveniles/adults of both sexes were included in the analyses.

### 4.2. Tissue Preparation for Histology

Animals were deeply anesthetised with 0.0016% tricaine methanesulfonate (MS-222, Sigma-Aldrich, St. Louis, MO, USA), euthanised, and fixed by immersion in 4% paraformaldehyde in 0.1 M phosphate-buffered saline pH 7.4 (PBS) for 2 h (from 2 to 7 dpf) and 1 day (from 1.5 mpf to 3–4 ypf) at 4 °C. After fixation, the lens was removed from the eye in specimens from 1.5 mpf onwards. Eyes were dissected out from the rest of the body in specimens from 8.5 mpf onwards. After rinsing in PBS, the animals or eyes were cryoprotected with 30 % sucrose in PBS, embedded in Neg-50TM (Thermo Scientific, Kalamazoo, MI, USA), and frozen with liquid nitrogen-cooled isopentane. Transverse sections (18 μm thick) were obtained on a cryostat and mounted on Superfrost Plus slides (Menzel-Glasser, Madison, WI, USA).

### 4.3. Haematoxylin–Eosin Staining

Some sections from 2 dpf and 1.5 mpf specimens were stained with haematoxylin–eosin following standard protocols. Briefly, cryostat sections were dried at room temperature (RT), rinsed in 0.05 M Tris-buffered (pH 7.4) saline (TBS) for 10 min, and stained with haematoxylin solution for 10 min. Sections were subsequently rinsed in tap water until removal of the excess of haematoxylin, in distilled water for 10 min, and then stained with eosin for 2 min. Finally, the sections were dehydrated and mounted in DPX mounting medium (Scharlau, Sentmenat, Spain).

### 4.4. Immunofluorescence

Sections were first pre-treated with 0.01 M citrate buffer pH 6.0 for 30 min at 90 °C for heat-induced epitope retrieval, allowed to cool for 20 min at RT, and rinsed in TBS for 5 min. Then, sections were incubated overnight at RT with a combination of two primary antibodies: a mouse monoclonal anti-PCNA (1:500; Sigma-Aldrich; catalogue number P8825; RRID: AB_477413) and a rabbit polyclonal anti-pH3) (1:300; Millipore; Billerica, MA, USA; catalogue number 06–570; RRID: AB_310177). Sections were rinsed 3 times in TBS for 10 min each and incubated for 1 h at RT with a combination of fluorescent dye-labelled secondary antibodies: a Cy3-conjugated goat anti-rabbit (1:200; Invitrogen, Waltham, MA, USA; catalogue number A10520) and a FITC-conjugated goat anti-mouse (1:200; Invitrogen; catalogue number F2761). All antibody dilutions were made in TBS containing 15% normal goat serum (Millipore), 0.2% Triton X-100 (Sigma-Aldrich), and 2% BSA (Sigma-Aldrich). Sections were then rinsed 3 times in TBS for 10 min each and in distilled water for 30 min, allowed to dry for 30 min at 37 °C, and mounted in MOWIOL^®^ 4–88 (Calbiochem, Darmstadt, Germany).

### 4.5. Specificity of Antibodies

Anti-PCNA antibodies (including the one used in this study) have been traditionally used to label proliferating cells in the retina of different fish (including zebrafish): *Oryzias latipes* [56]; *Haplochromis burtoni* [57,58]; *Onchorynchus mykiss* [59]; *Tinca tinca* [60,61,62]; *Carassius auratus* [56,61]; *Salmo trutta fario* [63]; *Danio rerio* [61,64]; *Scyliorhinus canicula* [38,65,66,67,68,69]; and *Petromyzon marinus* [37]. Anti-pH3 antibodies (including the one used in this study) have been also commonly used to label mitotic cells in the retina of fish (including zebrafish): *Carassius auratus* [49]; *Scyliorhinus canicula* [38,65,70]; and *Danio rerio* [71,72,73].

### 4.6. Image Acquisition

Brightfield images of haematoxylin–eosin-stained sections were taken with an Olympus BX51 microscope equipped with an Olympus DP71 camera. Images of fluorescent labelled sections were taken with a Leica TCS-SP2 confocal microscope with a combination of blue and green excitation lasers. Confocal optical sections were taken at steps of 1 μm along the *z*-axis. Collapsed images of the whole retinal sections (18 µm) were obtained with the LITE software (Leica, Wetzlar, Germany). For figure preparation, contrast and brightness of the images were minimally adjusted using Adobe Photoshop CS4 (Adobe, San Jose, CA, USA).

### 4.7. Cell Quantifications and Statistical Analyses

We quantified the number of mitotic cells (pH3+) in the whole retina (CMZ and central retina) and cells progressing through the cell cycle (PCNA+) in the central retina. As indicated above, the number of PCNA+ cells was not quantified in the CMZ because, at some stages (mainly 2 dpf), the high number of positive cells impeded to clearly differentiate between cells individually. In any case, the number of PCNA+ cells/section in the CMZ of adult zebrafish (6 to 48 mpf) was previously examined by Van Houcke et al. [40].

The numbers of pH3+ and PCNA+ cells were manually counted under a fluorescence microscope in one out of each two consecutive retinal sections (thickness of 18 μm). The limit between the CMZ and the central retina was established based on the expression pattern of PCNA, which is mainly found in the most peripheral region of the retina. In 2 dpf specimens, we did not separate pH3+ cell quantifications of the CMZ and central retina because, at this developmental stage, PCNA was highly expressed throughout the entire retina, which impeded to establish a clear limit between both regions. We calculated the mean number of cells per section for each retina. Then, we calculated the mean number of cells per retinal section for each animal based on the values for the two retinas and used that value for statistical analyses (each dot in the graph represents one animal). We also quantified the differential distribution of pH3+ and PCNA+ cells in the different cell layers of the central retina from 4 dpf onwards: the GCL, the INL, and the ONL.

Statistical analyses were performed with Prism 8 (GraphPad software, La Jolla, CA, USA). Normality of the data was determined with the Kolmogorov–Smirnov test. An ordinary one-way ANOVA followed by a Tukey’s multiple comparison test was used to determine statistically significant differences in normally distributed data. A Kruskal–Wallis test, followed by a Dunn’s multiple comparison test, was used to determine statistically significant differences in non-normally distributed data.

## 5. Conclusions

Our results reveal a decline in constitutive proliferative activity in the zebrafish retina with ageing. Importantly, mitotic activity is virtually absent in aged animals. Statements regarding the continuous and high proliferative activity in the fish (teleost) retina throughout life should be nuanced.

Interestingly, our systematic study also detected the presence of a secondary wave of cell proliferation during sexual maturation in the zebrafish retina. A possible relationship to the generation of the photoreceptors needed for sexual/adult behaviours is suggested. Future work should try to decipher the origin and destiny of these progenitor cells and their relationship to zebrafish behaviour.

## Data Availability

Data are contained within the article, and materials can be requested from the authors upon reasonable request.

## Supplementary Materials

The following are available online at https://www.mdpi.com/article/10.3390/ijms222111715/s1. Table S1: Studies demonstrating the presence of proliferating cells in the juvenile/adult retina of different teleost species. File S1: Mean ± S.E.M. data and data on statistical multiple comparisons related to graphs shown in Figure 2; File S2: Mean ± S.E.M. data and data on statistical multiple comparisons related to graphs shown in Figure 3.
