# Supplementary material for: Decline in Constitutive Proliferative Activity in the Zebrafish Retina with Ageing
Source: Int J Mol Sci. 2021 Oct 28;22(21):11715. doi: 10.3390/ijms222111715 (PMC8583983; doi:10.3390/ijms222111715)
Supplement: Supplementary file 1 [file ijms-22-11715-s001.zip › Table S1.pdf]

**Species**	**Stages/ages/sizes studied**	**Maximum size/age of specimens of each species**	**Method to label proliferating or mitotic cells**	**Retinal region of analysis**	**Quantification and comparison between different life stages or ages**	**Reference**
Haplochromis burtoni (African cichlid)	Juveniles and adults	12-15 cm	H^3-Thymidine	ONL	No (Qualitative descriptions: "dividing cells in the ONL were easier to demonstrate in younger fish")	[45]
	Adults (3.3 cm)	12-15 cm	BrdU and PCNA	ONL	No	[57]
	Adults (3 cm)	12-15 cm	PCNA	ONL	No	[74]
	Adults (3.5 cm)	12-15 cm	BrdU and PCNA	ONL	No	[58]
Carassius auratus (goldfish)	Juveniles (3-6 cm)	12-22 cm to 45 cm<br>30 years	Methyl- H3-Thymidine	Whole retina	No	[75]
	Juveniles and adults	12-22 cm to 45 cm<br>30 years	H3-Thymidine	ONL	No (Qualitative descriptions: "dividing cells in the ONL were easier to demonstrate in younger fish")	[45]

	Juveniles and adults (3-13 cm)	12-22 cm to 45 cm 30 years	H3-Thymidine	ONL	No	[48]
	Late larvae/early juveniles (20 to 51 days post-hatching)	12-22 cm to 45 cm 30 years	H3-Thymidine	INL and ONL	Yes (although no statistical analyses were used to compare different ages and only 1 or 2 sections were analysed per retina)	[76]
	Embryos, larvae [hatching (H0) and 1-14 days post-hatching] and adults	12-22 cm to 45 cm 30 years	PCNA	Whole retina	No	[56]
	Adults (2-6 cm)	12-22 cm to 45 cm 30 years	BrdU	ONL	No	[77]
	Juveniles (2.5-3.8 cm)	12-22 cm to 45 cm 30 years	BrdU and pH3	Whole retina	No	[49]
	Adults (9-12 cm)	12-22 cm to 45 cm 30 years	PCNA	Whole retina	No	[61]

Salmo trutta (trout)	Larvae (newly hatched and 3-6 weeks post-hatching), early (3 months post-hatching) and late (1-2 years post-hatching) juveniles	40-80 cm to 140 cm<br>2-3 years (sexual maturity)<br>8 to 20 years	Heidenhain's haematoxylin and eosin, Ehrlich's haematoxylin and eosin, and Mallory stainings	Whole retina	No	[78]
Onchorynchus mykiss (rainbow trout)	Early (2 months post-hatching) to late (2 years post-hatching) juveniles	3-6 mm (egg) to 12-20 mm (hatched)<br>2-3 years	PCNA, BrdU and IdU	INL	No (authors show that the density of PCNA+ cells in the INL decreases as eye diameter increases but without any statistical comparison)	[59]
Oryzias latipes (medaka)	Embryos, larvae [hatching (H0) and 1-14 days post-hatching] and adults	3-6 cm<br>2 to 3-5 years	PCNA	Whole retina	No	[56]
Tinca tinca (tench)	Adults	25-30 cm<br>20 years	PCNA	Whole retina	No	[60]
	Adults (13-16 cm)	25-30 cm<br>20 years	PCNA	Whole retina	No	[61]

Danio rerio (zebrafish)	Embryos (24 and 48 hpf) and adults (6-8 months postfertilization)	Sexual maturation (3 months) 2/3 to 5 years old	BrdU	Whole retina	No (qualitative descriptions: "the number of labelled cells is greater in the embryos")	[39]
	Adults (3.5-4 cm)	Sexual maturation (3 months) 2/3 to 5 years old	PCNA	Whole retina	No	[61]
	Juveniles (1 to 2 mpf)	Sexual maturation (3 months) 2/3 to 5 years old	BrdU and PCNA	Central retina	No	[17]
	Adults (6 to 48 mpf)	Sexual maturation (3 months) 2/3 to 5 years old	PCNA	CMZ	Yes (decreased proliferation with ageing)	[40]

References

1. Doetsch, F. A niche for adult neural stem cells. *Curr. Opin. Genet. Dev.* **2003**, 13(5), 543-550. doi: 10.1016/j.gde.2003.08.012.

2. Álvarez-Buylla, A.; Lim, D.A. For the long run: maintaining germinal niches in the adult brain. *Neuron*. **2004**, 41, 683-686. doi: 10.1016/S0896-6273(04)00111-4
3. Ferretti, P. Is there a relationship between adult neurogenesis and neuron generation following injury across evolution? *Eur. J. Neurosci*. **2011**, 34(6), 951-962. doi: 10.1111/j.1460-9568.2011.07833.x
4. Zupanc, G.K.; Sîrbulescu, R.F. Adult neurogenesis and neuronal regeneration in the central nervous system of teleost fish. *Eur. J. Neurosci*. **2011**, 34(6), 917-929. doi: 10.1111/j.1460-9568.2011.07854.x
5. Grandel, H.; Brand, M. Comparative aspects of adult neural stem cell activity in vertebrates. *Dev Genes Evol*. **2013**, 223(1-2), 131-147. doi: 10.1007/s00427-012-0425-5
6. Than-Trong, E.; Bally-Cuif, L. Radial glia and neural progenitors in the adult zebrafish central nervous system. *Glia*. **2015**, 63(8), 1406-1428. doi: 10.1002/glia.22856
7. Alunni, A.; Bally-Cuif, L. A comparative view of regenerative neurogenesis in vertebrates. *Development*. **2016**, 143(5), 741-753. doi: 10.1242/dev.122796
8. Zupanc, G.K.H. Adult neurogenesis in the central nervous system of teleost fish: from stem cells to function and evolution. *J. Exp. Biol*. **2021**, 224(8), jeb226357. doi: 10.1242/jeb.226357
9. Miles, A.; Tropepe, V. Retinal stem cell 'retirement plans': growth, regulation and species adaptations in the retinal ciliary marginal zone. *Int. J. Mol. Sci*. **2021**, 22, 6528. doi:10.3390/ijms22126528
10. Harris, W.A.; Perron, M. Molecular recapitulation: the growth of the vertebrate retina. *Int. J. Dev. Biol*. **1998**, 42(3), 299-304.
11. Fischer, A.J. Neural regeneration in the chick retina. *Prog. Retin. Eye Res*. **2005**, 24(2), 161-182. doi: 10.1016/j.preteyeres.2004.07.003
12. Raymond, P.A.; Barthel, L.K., Bernardos, R.I., Perkowski, J.J. Molecular characterization of retinal stem cells and their niches in adult zebrafish. *BMC Develop. Biol*. **2006**, 6, 36. doi: 10.1186/1471-213X-6-36
13. Fischer, A.J.; Bosse, J.L.; El-Hodiri, M. The ciliary marginal zone (CMZ) in development and regeneration of the vertebrate eye. *Exp. Eye Res*. **2013**, 116, 199-204. doi: 10.1016/j.exer.2013.08.018
14. Marcucci, F.; Murcia-Belmonte, V.; Wang, Q.; Coca, Y.; Ferreiro-Galve, S.; Kuwajima, T.; Khalid, S.; Ross, M.E.; Mason, C.; Herrera, E. The ciliary margin zone of the mammalian retina generates retinal ganglion cells. *Cell Rep*. **2016**, 17, 3153-3164. doi: 10.1016/j.celrep.2016.11.016
15. Bélanger, M.C.; Robert, B.; Cayouette, M. Msx1-positive progenitors in the retinal ciliary margin give rise to both neural and non-neural progenies in mammals. *Dev. Cell*. **2017**, 40(2), 137-150. doi: 10.1016/j.devcel.2016.11.020
16. Fausett, B.V.; Goldman, D. A role for α1 tubulin-expressing Müller glia in regeneration of the injured zebrafish retina. *J. Neurosci*. **2006**, 26(23), 6303-6313. doi: 10.1523/JNEUROSCI.0332-06.2006
17. Bernardos, R.L.; Barthel, L.K.; Meyers, J.R.; Raymond, P.A. Late-stage neuronal progenitors in the retina are radial Müller glia that function as retinal stem cells. *J. Neurosci*. **2007**, 27(26), 7028-7040. doi: 10.1523/JNEUROSCI.1624-07.200
18. Nagashima, M.; Barthel, L.K.; Raymond, P.A. A self-renewing division of zebrafish Müller glial cells generates neuronal progenitors that require N-cadherin to regenerate retinal neurons. *Development.* **2013**, 140, 4510-4521. doi: 10.1242/dev.090738
19. Wilken, M.S, Reh, T.A. Retinal regeneration in birds and mice. *Curr. Opin. Genet. Dev*. **2016**, 40, 57-64. doi: 10.1016/j.gde.2016.05.028
20. Okada, T.S. Cellular metaplasia or transdifferentiation as a model for retinal cell differentiation. *Curr. Top. Dev. Biol*. **1980**, 16, 349-380
21. Engelhardt, M.; Bogdahn, U.; Aigner, L. Adult retinal pigment epithelium cells express neural progenitor properties and the neuronal precursor protein doublecortin. *Brain Res*. **2005**, 1040(1-2), 98-111. doi: 10.1016/j.brainres.2005.01.075
22. Ma, R.T.Y.; Li, X.; Wang, S. Z. Reprogramming RPE to differentiate towards retinal neurons with Sox2. *Stem Cells*. **2009**, 27, 1376-1387. doi: 10.1002/stem.48
23. Eymann, J.; Salomies, L.; Macrì, S.; Di-Poï, N. Variations in the proliferative activity of the peripheral retina correlate with postnatal ocular growth in squamate reptiles. *J. Comp. Neurol*. **2019**, 527, 2356-2370. doi: 10.1002/cne.24677

24. Tropepe, V.; Coles, B.L.; Chiasson, B.J.; Horsford, D.J.; Elia, A.J.; McInnes, R.R.; van der Kooy, D. Retinal stem cells in the adult mammalian eye. *Science*. **2000**, 287(5460), 2032-2036. doi: 10.1126/science.287.5460.2032
25. Fischer, A.J.; Reh, T.A. Transdifferentiation of pigmented epithelial cells: a source of retinal stem cells? *Dev. Neurosci*. **2001**, 23(4-5), 268-276. doi: 10.1159/000048710
26. Fischer, A.J; Reh, T.A. Growth factors induce neurogenesis in the ciliary body. *Dev. Biol*. **2003**, 259(2), 225-240. doi: 10.1016/s0012-1606(03)00178-7
27. Das, A.V.; James, J.; Rahnenführer, J.; Thoreson, W.B.; Bhattacharya, S.; Zhao, X.; Ahmad, I. Retinal properties and po-tential of the adult mammalian ciliary epithelium stem cells. *Vision Res*. **2005**, 45(13), 1653-1666. doi: 10.1016/j.visres.2004.12.017
28. Das, A.V.; Zhao, X.; James, J.; Kim, M.; Cowan, K.H.; Ahmad, I. Neural stem cells in the adult ciliary epithelium express GF.A.P and are regulated by Wnt signaling. *Biochem. Biophys. Res. Commun*. **2006**, 339(2), 708-716. doi: 10.1016/j.bbrc.2005.11.064
29. Reh, T.A.; Fischer, A.J. Stem cells in the vertebrate retina. *Brain Behav. Evol*. **2001**, 58(5), 296-305. doi: 10.1159/000057571
30. Amato, M.A.; Arnault, E.; Perron, M. Retinal stem cells in vertebrates: Parallels and divergences. *Int. J. Dev. Biol*. **2004**, 48, 993-1001. doi: 10.1387/ijdb.041879ma
31. Moshiri, A.; Close, J.; Reh, T.A. Retinal stem cells and regeneration. *Int. J. Dev. Biol*. **2004**, 48, 1003-1014. doi: 10.1387/ijdb.041870am
32. Fernald, R.D. Teleost vision: seeing while growing. *J. Exp. Zool*. **1991**, Suppl. 5, 167-180. doi: 10.1002/jez.1402560521
33. Perron, M.; Harris, W.A. Retinal stem cells in vertebrates. *Bioessays*. **2000**, 22(8), 685-688. doi: 10.1002/1521-1878(200008)22:8<685::AID-BIES1>3.0.CO;2-C
34. Kubota, R.; Hokoc, J.N.; Moshiri, A.; McGuire, C.; Reh, T.A. A comparative study of neurogenesis in the retinal ciliary marginal zone of homeothermic vertebrates. *Dev. Brain Res*. **2002**, 134, 31-41. doi: 10.1016/S0165-3806(01)00287-5
35. Wan, Y.; Almeida, A.D.; Rulands, S.; Chalour, N.; Muresan, L.; Wu., Y.; Simons, B.D.; He, J.; Harris, W.A. The ciliary marginal zone of the zebrafish retina: clonal and time-lapse analysis of a continuously growing tissue. *Development*. **2016**, 143, 1099-1107. doi: 10.1242/dev.133314
36. García-Pradas, L.; Gleiser, C.; Wizenmann, A.; Wolburg, H.; Mack, A.F. Glial cells in the fish retinal nerve fiber layer form tight junctions, separating and surrounding axons. *Front. Mol. Neurosci*. **2018**, 11, 367. doi: 10.3389/fnmol.2018.00367
37. Villar-Cheda, B.; Abalo, X.M.; Villar-Cerviño, V.; Barreiro-Iglesias, A.; Anadón, R.; Rodicio, M.C. Late proliferation and photoreceptor differentiation in the transforming lamprey retina. *Brain Res*. **2008**, 1201, 60-67. doi: 10.1016/j.brainres.2008.01.077
38. Hernández-Núñez, I.; Robledo, D.; Mayeur, H.; Mazan, S.; Sánchez, L.; Adrio, F.; Barreiro-Iglesias, A.; Candal E. Loss of active neurogenesis in the adult shark retina. *Front. Cell Dev. Biol*. **2021**, 9, 628721. doi: 10.3389/fcell.2021.628721
39. Marcus, R.C.; Delaney, C.L; Easter, S.S. Jr. Neurogenesis in the visual system of embryonic and adult zebrafish (*Danio rerio*). off. *Vis. Neurosci*. **1999**, 16(3), 417-424. doi: 10.1017/s095252389916303x
40. Van Houcke, J.; Geeraerts, E.; Vanhunsel, S.; Beckers, A.; Noterdaeme, L.; Christiaens, M.; Bollaerts; I., De Groef, L.; Moons, L. Extensive growth is followed by neurodegenerative pathology in the continuously expanding adult zebrafish retina. *Biogerontology*. **2019**, 20(1), 109-125. doi: 10.1007/s10522-018-9780-6
41. Mandyam, C.D; Harburg, G.C.; Eisch, A.J. Determination of key aspects of precursor cell proliferation, cell cycle length and kinetics in the adult mouse subgranular zone. *Neuroscience*. **2007**, 146(1), 108-122. doi: 10.1016/j.neuroscience.2006.12.064
42. Malicki, J.; Neuhauss, S.C.; Schier, A.F.; Solnica-Krezel, L.; Stemple, D.L.; Stainier, D.Y.; Abdelilah, S.; Zwartkruis, F.; Rangini, Z.; Driever, W. Mutations affecting development of the zebrafish retina. *Development*. **1996**, 123, 263-273.
43. Easter, S.S., Jr.; Nicola, G.N. The development of vision in the zebrafish (*Danio rerio*). *Dev. Biol*. **1996**, 180, 646-663. doi: 10.1006/dbio.1996.0335
44. Zerjatke, T.; Gak, I.A.; Kirova, D.; Fuhrmann, M.; Daniel, K.; Gonciarz, M.; Müller, D.; Glauche, I.; Mansfeld, J. Quantitative cell cycle analysis based on an endogenous all-in-one reporter for cell tracking and classification. *Cell Rep*. **2017**, 19(9), 1953-1966. doi: 10.1016/j.celrep.2017.05.022
45. Johns, P.R.; Fernald, R.D. Genesis of rods in teleost fish retina. *Nature*. **1981**, 293(5828), 141-142. doi: 10.1038/293141a0.

46. Biehlmaier, O.; Neuhauss, S.C.; Kohler, K. Onset and time course of apoptosis in the developing zebrafish retina. *Cell Tissue Res*. **2001**, 306(2), 199-207. doi: 10.1007/s004410100447
47. Li, L.; Wojtowicz, J.L.; Malin, J.H.; Huang, T.; Lee, E.B.; Chen, Z. GnRH-mediated olfactory and visual inputs promote mating-like behaviors in male zebrafish. *PLoS One*. **2017**, 12(3), e0174143. doi: 10.1371/journal.pone.0174143
48. Johns, P.R. Formation of photoreceptors in larval and adult goldfish. *J. Neurosci.* **1982**, 2(2), 178-198. doi: 10.1523/JNEUROSCI.02-02-00178.1982
49. Otteson, D.C; D'Costa, A.R.; Hitchcock, P.F. Putative stem cells and the lineage of rod photoreceptors in the mature retina of the goldfish. *Dev. Biol.* **2001**, 232(1), 62-76. doi: 10.1006/dbio.2001.0163
50. Morris, A.C.; Scholz, T.L.; Brockerhoff, S.E.; Fadool, J.M. Genetic dissection reveals two separate pathways for rod and cone regeneration in the teleost retina. *Dev. Neurobiol*, **2008a**, 68(5), 605-619. doi: 10.1002/dneu.20610
51. Morris, A.C.; Scholz, T.; Fadool, J.M. Rod progenitor cells in the mature zebrafish retina. *Adv. Exp. Med. Biol.*, **2008b**, 613, 361-368. doi: 10.1007/978-0-387-74904-4_42
52. Lenkowski, J.R.; Raymond, P.A. Müller glia: stem cells for generation and regeneration of retinal neurons in teleost fish. *Prog. Retin. Eye Res*. **2014**, 40, 94-123. doi: 10.1016/j.preteyeres.2013.12.007
53. Stenkamp D.L. The rod photoreceptor lineage of teleost fish. *Prog. Retin. Eye Res*., **2011**, 30(6), 395-404. doi:10.1016/j.preteyeres.2011.06.004
54. Crespo, C.; Knust, E. Characterisation of maturation of photoreceptor cell subtypes during zebrafish retinal development. *Biol. Open*. **2018**, 7(11), bio036632. doi: 10.1242/bio.036632
55. Hutter, S.; Hettyey, A.; Penn, D.J.; Zala, S.M. Ephemeral sexual dichromatism in zebrafish (*Danio rerio*). *Ethology*. **2012**, 118, 1208-1218. doi: 10.1111/eth.12027
56. Negishi, K.; Stell, W.K.; Takasaki, Y. Early histogenesis of the teleostean retina: studies using a novel immunochemical marker, proliferating cell nuclear antigen (PCNA/cyclin). *Brain Res. Dev*. **1990**, 55(1), 121-125.
57. Mack, A.F.; Fernald, R.D. New rods move before differentiating in adult teleost retina. *Dev. Biol.* **1995**, 170(1), 136-141. doi: 10.1006/dbio.1995.1202
58. Mack, A.F.; Fernald, R.D. Cell movement and cell cycle dynamics in the retina of the adult teleost *Haplochromis burtoni*. *J. Comp. Neurol*. **1997**, 388(3), 435-443.
59. Julian, D.; Ennis, K.; Korenbrot, J.I. Birth and fate of proliferative cells in the inner nuclear layer of the mature fish retina. *J. Comp. Neurol*. **1998**, 394(3), 271-282.
60. Velasco, A.; Cid, E.; Ciudad, J.; Orfao, A.; Aijón, J.; Lara, J.M. Temperature induces variations in the retinal cell proliferation rate in a cyprinid. *Brain Res*. **2001**, 913, 190-194. doi:10.1016/S0006-8993(01)02804-9
61. Cid, E.; Velasco, A.; Ciudad, J.; Orfao, A.; Aijón, J.; Lara, J.M. Quantitative evaluation of the distribution of proliferating cells in the adult retina in three cyprinid species. *Cell Tissue Res.* **2002**, 308, 47-59. doi: 10.1007/s00441-002-0529-8
62. Jimeno, D.; Lillo, C.; Cid, E.; Aijón, J.; Velasco, A.; Lara, J.M. The degenerative and regenerative processes after the elim-ination of the proliferative peripheral retina of fish. *Exp. Neurol*. **2003**, 179(2), 210-228. doi: 10.1016/S0014-4886(02)00020-1
63. Candal, E.; Anadón, R.; DeGrip, W.J.; Rodríguez-Moldes, I. Patterns of cell proliferation and cell death in the developing retina and optic tectum of the brown trout. *Brain Res. Dev*. **2005**, 154(1), 101-119. doi: 10.1016/j.devbrainres.2004.10.008
64. Amini, R.; Labudina, A.A.; Norden, C. Stochastic single cell migration leads to robust horizontal cell layer formation in the vertebrate retina. *Development*. **2019**, 146(12), dev173450. doi: 10.1242/dev.173450
65. Ferreiro-Galve, S.; Rodríguez-Moldes, I.; Anadón, R.; Candal, E. Patterns of cell proliferation and rod photoreceptor differentiation in shark retinas. *J. Chem. Neuroanat*. **2010**, 39, 1-14. doi: 10.1016/j.jchemneu.2009. 10.001
66. Ferreiro-Galve, S.; Rodríguez-Moldes, I.; Candal, E. Calretinin immunoreactivity in the developing retina of sharks: comparison with cell proliferation and GABAergic system markers. *Exp. Eye Res*. **2010**, 91, 378-386. doi: 10.1016/j.exer.2010.06.011

67. Ferreiro-Galve, S.; Rodríguez-Moldes, I.; Candal, E. Pax6 expression during retinogenesis in sharks: comparison with markers of cell proliferation and neuronal differentiation. *J. Exp. Zool. B Mol. Dev. Evol*. **2012**, 318, 91-108. doi: 10.1002/jezb.21448
68. Sánchez-Farías, N.; Candal, E. Doublecortin widely expressed in the developing and adult retina of sharks. *Exp. Eye Res*. **2015**, 134, 90-100. doi: 10.1016/j.exer.2015.04.002
69. Sánchez-Farías, N.; Candal, E. Identification of radial glia progenitors in the developing and adult retina of sharks. *Front. Neuroanat.* **2016**, 10, 65. doi: 10.3389/fnana.2016.00065
70. Bejarano-Escobar, R.; Blasco, M.; Durán, A.C.; Rodríguez, C.; Martín-Partido, G.; Francisco-Morcillo, J. Retinal histogenesis and cell differentiation in an elasmobranch species, the small-spotted catshark *Scyliorhinus canicula*. *J. Anat.* **2012**, 220, 318-335. doi: 10.1111/j.1469-7580.2012.01480.x
71. Jensen, A.M.; Walker, C.; Westerfield, M. Mosaic eyes: a zebrafish gene required in pigmented epithelium for apical localization of retinal cell division and lamination. *Development*. **2001**, 128(1), 95-105.
72. Godinho, L.; Williams, P.R.; Claassen, Y.; Provost, E.; Leach, S.D.; Kamermans, M.; Wong, R.O. Nonapical symmetric divisions underlie horizontal cell layer formation in the developing retina in vivo. *Neuron*. **2007**, 56(4), 597-603. doi: 10.1016/j.neuron.2007.09.036
73. Weber, I.P.; Ramos, A.P.; Strzyz, P.J.; Leung, L.C.; Young, S.; Norden, C. Mitotic position and morphology of committed precursor cells in the zebrafish retina adapt to architectural changes upon tissue maturation. *Cell Rep*. **2014**, 7(2), 386-397. doi: 10.1016/j.celrep.2014.03.014
74. Kwan, J.W.; Lee M.J.; Mack A.F.; Chiu J.F.; Fernald R.D. Nonuniform distribution of cell proliferation in the adult teleost retina. *Brain Res*. **1996**, 712, 40-44. doi: 10.1016/0006-8993(95)01426-8
75. Meyer, R.L. Evidence from thymidine labeling for continuing growth of retina and tectum in juvenile goldfish. *Exp. Neurol*. **1978**, 59, 99-111. doi: 10.1016/0014-4886(78)90204-2
76. Raymond, P.A.; Rivlin, P.K. Germinal cells in the goldfish retina that produce rod photoreceptors. *Dev. Biol.* **1987**, 122(1), 120-138. doi: 10.1016/0012-1606(87)90338-1
77. Stenkamp, D.L.; Barthel, L.K.; Raymond, P. A. Spatiotemporal coordination of rod and cone photoreceptor differentiation in goldfish retina. *J. Comp. Neurol*, **1997**. 382(2), 272-284. doi: 10.1002/(sici)1096-9861(19970602)382:2<272::aid-cne10>3.0.co;2-u
78. Lyall, A.H. The growth of the trout retina. *Q. J. Micros. Sci.* **1957**, 98, 101-110.
